# Proteomic Analysis of Chicken Skeletal Muscle during Embryonic Development

**DOI:** 10.3389/fphys.2017.00281

**Published:** 2017-05-08

**Authors:** Hongjia Ouyang, Zhijun Wang, Xiaolan Chen, Jiao Yu, Zhenhui Li, Qinghua Nie

**Affiliations:** ^1^Department of Animal Genetics, Breeding and Reproduction, College of Animal Science, South China Agricultural UniversityGuangzhou, China; ^2^Guangdong Provincial Key Lab of Agro-Animal Genomics and Molecular Breeding, and Key Lab of Chicken Genetics, Breeding and Reproduction, Ministry of AgricultureGuangzhou, China

**Keywords:** proteome, chicken, skeletal muscle, embryonic development, iTRAQ

## Abstract

Embryonic growth and development of skeletal muscle is a major determinant of muscle mass, and has a significant effect on meat production in chicken. To assess the protein expression profiles during embryonic skeletal muscle development, we performed a proteomics analysis using isobaric tags for relative and absolute quantification (iTRAQ) in leg muscle tissues of female Xinghua chicken at embryonic age (E) 11, E16, and 1-day post hatch (D1). We identified 3,240 proteins in chicken embryonic muscle and 491 of them were differentially expressed (fold change ≥ 1.5 or ≤ 0.666 and *p* < 0.05). There were 19 up- and 32 down-regulated proteins in E11 vs. E16 group, 238 up- and 227 down-regulated proteins in E11 vs. D1 group, and 13 up- and 5 down-regulated proteins in E16 vs. D1 group. Protein interaction network analyses indicated that these differentially expressed proteins were mainly involved in the pathway of protein synthesis, muscle contraction, and oxidative phosphorylation. Integrative analysis of proteome and our previous transcriptome data found 189 differentially expressed proteins that correlated with their mRNA level. The interactions between these proteins were also involved in muscle contraction and oxidative phosphorylation pathways. The lncRNA-protein interaction network found four proteins DMD, MYL3, TNNI2, and TNNT3 that are all involved in muscle contraction and may be lncRNA regulated. These results provide several candidate genes for further investigation into the molecular mechanisms of chicken embryonic muscle development, and enable us to better understanding their regulation networks and biochemical pathways.

## Introduction

Skeletal muscle is a highly complex and heterogeneous tissue, and serves several important metabolic functions (Bentzinger et al., 2012). In domestic animal, skeletal muscle is the largest contributor to mass and is therefore directly related to meat production (Güeller and Russell, 2010). Muscle mass depends on the number of muscle fibers and muscle hypertrophy. The number of muscle fiber is set at the time of birth, and this limitation can only be partially compensated by muscle hypertrophy after birth (Wigmore and Stickland, 1983; Ylihärsilä et al., 2007). Thus, embryonic myogenesis is a crucial process for increasing muscle mass.

Myogenesis is the formation of muscle fibers from somites and is highly regulated by many genes, transcription factors or non-coding RNAs (Buckingham, 2001; Bassel-Duby and Olson, 2006; Cesana et al., 2011). In chicken, skeletal muscle form in the limb from progenitor cells during embryogenesis. These progenitor cells are originated from somites and migrate into the limb bud. Subsequently, they proliferate and differentiate into skeletal muscle in limb (Buckingham et al., 2003). Numerous regulatory genes involved in myogenesis have been identified, such as Myf5 (myogenic factor 5), MyoD (myogenic differentiation 1), Mrf4 (myogenic regulatory factor 4) MyoG (myogenin), MEF2 (myocyte enhancer binding factor 2), Myostatin, IGF2 (insulin-like growth factors 2), PAX3 (paired box 3), and PAX7 (paired box 7) (Bismuth and Relaix, 2010; Braun and Gautel, 2011). However, the regulations of skeletal muscle development in the chicken are far from clear, more genes or proteins, and non-coding RNAs and their interactions need to further elucidate. Our previous study identified differentially expressed chicken mRNAs and long non-coding RNAs (lncRNA) during embryonic skeletal muscle development, and provides a functional interaction network between lncRNAs and protein-coding genes (Li et al., 2017). However, proteins and mRNA levels do not often directly correlate and proteins also provides post-translational modified information and more direct evidence (Greenbaum et al., 2003; Zhan et al., 2016). Thus, we aimed to assess the protein expression profiles during embryonic skeletal muscle development for this study.

The iTRAQ is a reliable and accurate technique for quantitative analysis in proteomics study (Wiese, 2007). This technique uses stable isotopically-labeled molecules that can be covalently bonded to the N-terminus and side chain amines of proteins, and have been increasingly applied to investigate the proteome in different organisms (Liu et al., 2016a; Minjarez et al., 2016; Xiong et al., 2016; Campos et al., 2017). In this study, we performed iTRAQ-based proteomics analysis using embryonic muscle samples, and identified differentially expressed proteins during skeletal muscle development in chickens. We further combined analysis this proteome data with our previous RNA sequencing to identify proteins whose expression directly correlated with mRNA as well as lncRNA expression patterns (Li et al., 2017).

## Materials and methods

### Ethics statement

Animal experiments were carried out in compliance with animal care protocols and all efforts were made to minimize suffering. The protocol was approved by the Animal Care Committee of South China Agricultural University (Guangzhou, China) with approval number SCAU#0014.

### Animals and samples preparation

Xinghua (XH) chickens at E10 were obtained from the Chicken Breeding Farm of South China Agricultural University (Guangzhou, China) and incubated in Automatic Incubator (Oscilla, Shandong, China) at 37.8°C, 50–70% relative humidity. Chicken embryo sexes were identified by PCR amplification of the *CHD*1 (chromodomain helicase DNA binding protein 1) gene (Fridolfsson and Ellegren, 1999). Leg muscles of female Xinghua chickens in three different development stages (E11, E16, and D1) were used for iTRAQ analysis.

### iTRAQ assays

Two female chickens of each stage E11, E16, and D1 were used for iTRAQ assays (Applied Biosystems, Foster city, CA, USA). Total proteins were extracted by using a urea lysis buffer (7 M urea, 2 M thiourea, and 1% SDS) containing 1 mM PMSF. Proteins were quantified using a BCA Assay Kit (Pierce, Thermo, USA) and detected by SDS-electrophoresis. Total proteins were treated follow reduction, cysteine alkylation, and trypsin digestion to obtain peptides, and then equal amounts of peptides from six samples were labeled individually with different iTRAQ reagents (E11, labeled by 113 and 114; E16, labeled by 115 and 116; and D1, labeled by 119 and 121) using instructions provided by the manufacturer. After iTRAQ-labeling, peptides were desalted using a C18 solid-phase extraction and submitted for Nano Liquid Chromatography–Mass Spectrometry/Mass Spectrometry (LC-MS/MS) analysis.

The experiments were performed on a Nano Aquity UPLC system (Waters Corporation, Milford, MA) connected to a quadrupole-Orbitrap mass Spectrometer (Q-Exactive) (Thermo Fisher Scientific, Bremen, Germany) equipped with an online nano-electrospray ion source. The Q-Exactive mass spectrometer was operated in the data-dependent mode to switch automatically between MS and MS/MS acquisition. Survey full-scan MS spectra (m/z 350–1,200) were acquired with a mass resolution of 70K, followed by fifteen sequential high energy collisional dissociation MS/MS scans with a resolution of 17.5K. In all cases, one microscan was recorded using dynamic exclusion of 60 s. MS/MS fixed first mass was set at 100.

### iTRAQ data analysis

The MS/MS data were analyzed with Proteome Discoverer software v1.4 (Thermo Scientific), and search in the Uniprot database (*Gallus gallus*). The target-decoy based strategy was applied to control peptide level false discovery rates (FDR) lower than 1%. Only unique peptides were used for protein quantification and normalization on protein medians was used to correct experimental bias, the minimum number of proteins that must be observed to allow was set to 200. For protein quantitation, the ratios of each sample were weighted and normalized by comparing the control group (sample tagged as 113) as the denominator. For quantitative changes, we set a >1.5 or <0.66-fold change cutoff and *p*-value (*t*-test) <0.05 for differentially expressed proteins.

All identified proteins were annotated and classified by GO (Gene Ontology, http://www.geneontology.org/) and KEGG (Kyoto Encyclopedia of Genes and Genomes, http://www.genome.jp/kegg/) pathway. The differentially expressed proteins were further processed by DAVID 6.8 Functional Annotation Tool (http://david.abcc.ncifcrf.gov/) for term enrichment analysis (Huang et al., 2009a). The results were filtered based on a Fisher Exact statistic methodology as previously described (Huang et al., 2009b). The GO biological network was assessed using the ClueGO of Cytoscape software (http://www.cytoscape.org/). Protein-protein interaction analysis was performance by String v10.0 (http://www.string-db.org/), and a high coefficient value of 0.7 was used as a cutoff (Szklarczyk et al., 2015). Cluster analysis was performed to identify the expression patterns of differentially expressed proteins (fold change ≥ 1.2 or ≤ 0.8 and *p* < 0.05) using hcluster (https://pypi.python.org/pypi/hcluster/0.2.0).

### Integrative analysis of the proteome and transcriptomes data

Transcriptomes data (including mRNAs and lncRNAs) obtained from our previous study were using for integrative analysis with the proteome data (Li et al., 2017). Differentially expressed proteins were compared with the differentially expressed mRNAs, to identify proteins that were consistently expressed at the RNA and protein levels. The correlation between these consistently expressed proteins was analyzed by String v10.0 with a coefficient value of 0.7. The differentially expressed proteins were also compared with the predicted potential target genes of differentially expressed lncRNAs. The interaction network of the corresponding proteins and lncRNAs were constructed by String v10.0 and Cytoscape software.

### Western blotting

Proteins were extracted from muscle tissues by using a urea lysis buffer (7 M urea, 2 M thiourea and 1% SDS) containing 1 mM PMSF. Total proteins (50 μg) were separated on a 12% SDS-PAGE, and transferred to a polyvinylidene difluoride membrane (Millipore, Bedford, MA). The membrane was blocked in 5% BSA blocking solution for 1 h at room temperature and incubated overnight at 4°C with primary antibodies (Abcam, Cambridge, UK) as follows: MYL1 (ab97427, diluted 1:500), MYL3 (ab137767, diluted 1:1,000), RPL4 (ab154907, diluted 1:1,000), STMN1(ab194670, diluted 1:1,000), RPS3A (ab101690, diluted 1:500), and TNNT3 (ab82784, diluted 1:500). After that, the membrane was washed with PBS-T and then developed with anti-mouse or rabbit horseradish peroxidase conjugated secondary antibodies (Sigma-Aldrich, diluted 1:5,000). Protein bands were visualized using enhanced chemiluminescence (ECL) system (GE Healthcare, USA) and quantified with an ImageQuant LAS4000 system (Fujifilm, Tokyo, Japan).

## Results

### Proteomic expression profiling of embryonic muscle in chickens

Embryonic growth and development of skeletal muscle has a significant effect on muscle mass in chickens (Halevy et al., 2004, 2006). To assess the protein expression profiles during embryonic skeletal muscle development, we used the same samples as our previous RNA sequencing (leg muscle tissues of female XH chicken at E11, E16, and D1; each stage two individuals) for iTRAQ-based proteomics analysis (Li et al., 2017).

**Figure 1:**
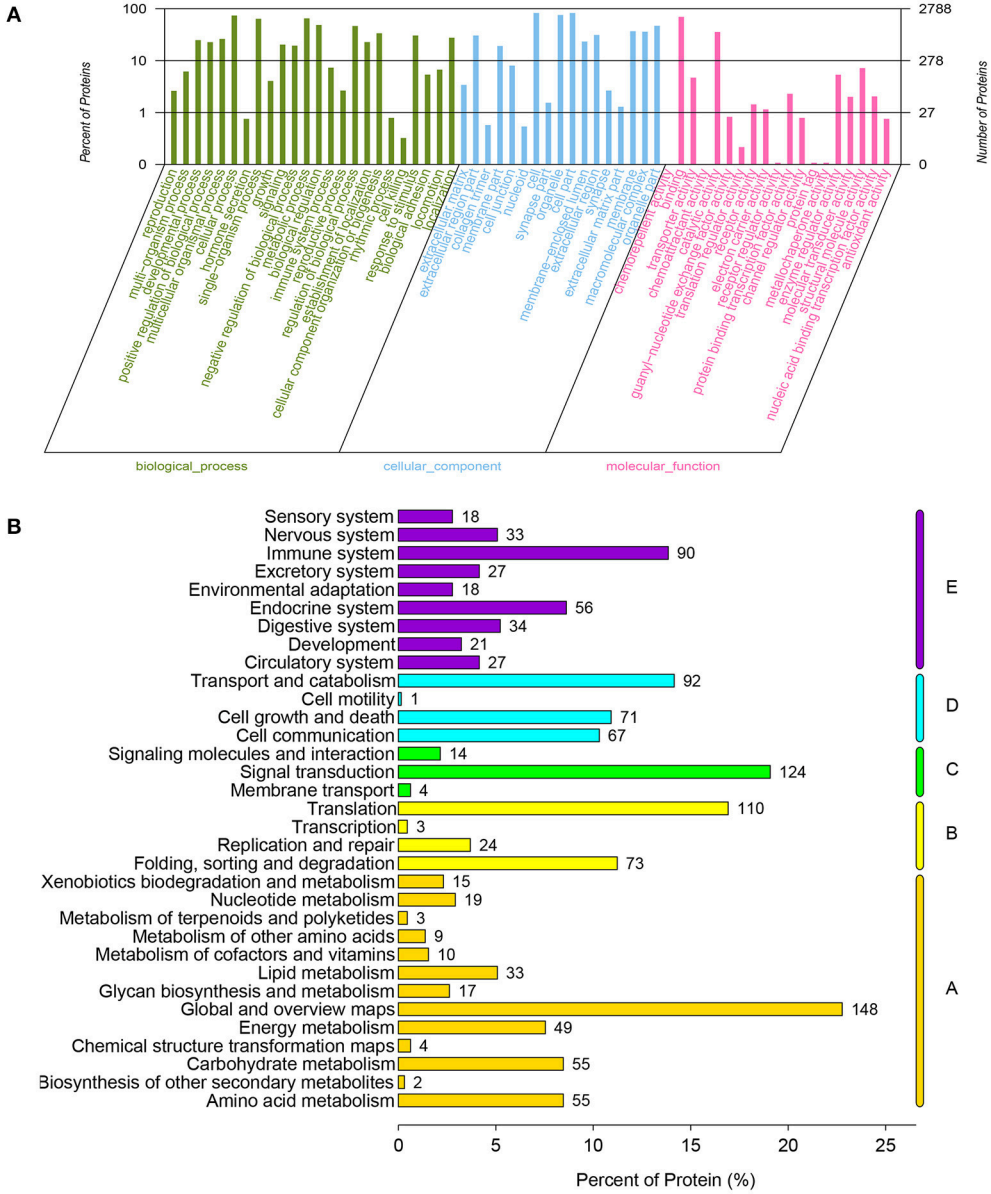
**Functional classification of all annotated proteins in chicken embryonic muscle. (A)** Proteins as classified into three main categories by GO analysis: biological process, cellular component, and molecular function. The left y-axis indicates the percentage of a specific category of genes in that category. The right y-axis indicates the number of genes in a category. **(B)** Proteins as classified into five main categories by KEGG analysis: metabolism, genetic information processing, environmental information processing, cellular processes and organismal systems. The x-axis indicates the percentage genes within that specific category.

The LC-MS/MS data analysis generated a total of 58,815 match spectra, 19,659 peptides, and 15,495 unique peptides. We could identify 3,240 proteins possessing at least one unique peptide with a confidence level above 95% (Table S1). All proteins were grouped according to biological process, cellular component, and molecular function by GO analysis (Table S2). The cellular process, metabolic process and biological regulation were mainly categories for these proteins in biological processes, and the binding and catalytic activity were the two most abundant categories in molecular function (Figure 1A). The KEGG analysis indicated that these proteins were primarily involved in pathway of signal transduction, translation, and transport and catabolism (Figure 1B).

### Identification of differentially expressed proteins

**Figure 2:**
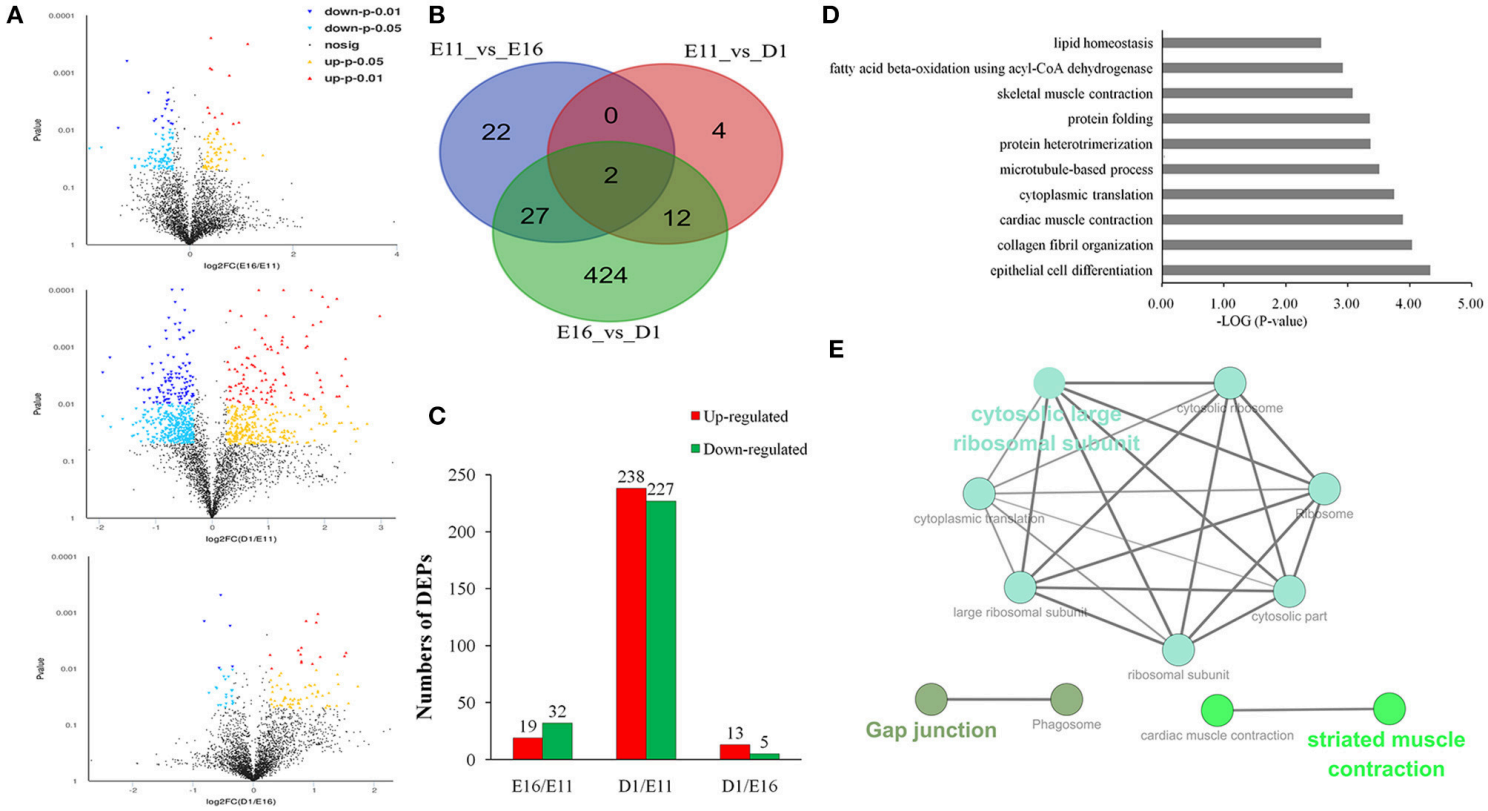
**Differentially expressed proteins in three different development stages of embryonic muscle. (A)** Volcano plots of differentially expressed proteins in three comparisons: E11 vs. E16, E11 vs. D1, and E16 vs. D1. **(B)** Venn diagrams of differentially expressed proteins (*n* = 491; *P* < 0.05, fold change > 1.5 or < 0.66). **(C)** Number of differentially expressed proteins in three comparisons: E11 vs. E16, E11 vs. D1, and E16 vs. D1. **(D)** The top 10 GO enrichment terms for differentially expressed proteins. **(E)** The 57 proteins in the top 10 biological processes were analysis using ClueGO of Cytoscape software.

To access proteins dynamic changes during embryonic development of skeletal muscle, we identified 491 differentially expressed proteins (fold change ≥ 1.5 or ≤ 0.666 and *p* < 0.05) from the three different developmental groups (Figure 2A). There were 19 up- and 32 down-regulated proteins in E11 vs. E16 (Table S3) group, 238 up- and 227 down-regulated proteins in E11 vs. D1 (Table S4) group, and 13 up- and 5 downregulated proteins in E16 vs. D1 (Table S5) group (Figures 2B,C). According to the *p*-values, the top 10 differentially expressed proteins of each comparison group were listed in Table 1. These differentially expressed proteins could play an important role on development of embryonic muscle, and served as candidate genes for further study.

**Table 1:** **The top 10 differentially expressed proteins in E11 vs. E16, E11 vs. D1, and E16 vs. D1 groups**.

**Accession number**	**Gene symbol**	**Protein name**	**Fold change**	***P*-value**
**E11 vs. E16**				
F1NLZ4	FMOD	Fibromodulin	2.187	0.0003
E1C1Z5	CCDC102B	Coiled-coil domain containing 102B	0.431	0.0006
E1C8N1	COMP	Cartilage oligomeric matrix protein	1.707	0.0011
F1P0A2	CTHRC1	Collagen triple helix repeat containing 1	0.580	0.0023
P68139	ACTA1	Actin alpha 1	1.539	0.0051
F2Z4L5	RPL7A	60S Ribosomal protein L7a	0.633	0.0066
E1C9A0	NASP	Nuclear autoantigenic sperm protein	0.620	0.0070
Q92007	N/A	Aldolase A	1.927	0.0074
F1P1G5	CECR5L	Cat eye syndrome chromosome region, candidate 5-like	1.793	0.0078
R4GL88	CRABP2	Cellular retinoic acid binding protein 2	0.383	0.0093
**E11 vs. D1**				
F1P360	CKAP4	Cytoskeleton associated protein 4	0.609	0.00003
F1P310	COQ9	Coenzyme Q9	2.423	0.00009
P02588	TNNC2	Troponin C, skeletal muscle	3.400	0.00009
E1BS96	ABLIM1	Actin-binding LIM protein 1	1.779	0.00010
A4UNW1	MYL10	Myosin light chain 10	3.897	0.00013
P02604	MYL1	Myosin light chain 1	4.668	0.00014
F1NWF2	JPH1	Junctophilin 1	0.633	0.00016
Q90885	N/A	Uncharacterized protein	4.190	0.00020
F1NP23	COL6A2	Collagen, type VI, alpha 2	1.948	0.00028
I0IUP3	MCM8	DNA helicase MCM8	7.932	0.00029
**E16 vs. D1**				
P16527	MARCKS	Myristoylated alanine rich protein kinase C substrate	0.568	0.0014
P80026	PVALB	Parvalbumin	2.912	0.0052
P07322	ENO3	Enolase 3	2.854	0.0060
P67881	CYCS	Cytochrome c	3.323	0.0206
Q9DEA3	PCNA	Proliferating cell nuclear antigen	0.654	0.0218
Q45KQ2	RBM3	RNA binding motif protein 3	0.657	0.0227
E1BR10	PRDX3	Peroxiredoxin 3	1.537	0.0241
E1C8T8	N/A	Uncharacterized protein	0.601	0.0273
F1P5R8	SMYD1	SET and MYND domain containing 1	1.529	0.0347
E1BT53	N/A	Uncharacterized protein	1.570	0.0367

### Function analysis of proteins in chicken embryonic muscle

In order to gain insights into the functions of these differentially expressed proteins, GO functional enrichment analysis was performed using DAVID tool. In total, 420 proteins were enriched into130 GO term. These were involved in epithelial cell differentiation, collagen fibril organization, muscle contraction, cytoplasmic translation and protein folding (Figure 2D). We also found significant enrichments for phagosome, ribosome, pyruvate metabolism, and Glycolysis/Gluconeogenesis by KEGG pathway analysis (Figure S1). We further analyzed 57 proteins taken from the top 10 biological processes and formed a functional interaction network. This analysis indicated that these significantly altered proteins were involved in structural constituent of ribosome as well as cardiac and striated muscle contraction (Figure 2E).

**Figure 3:**
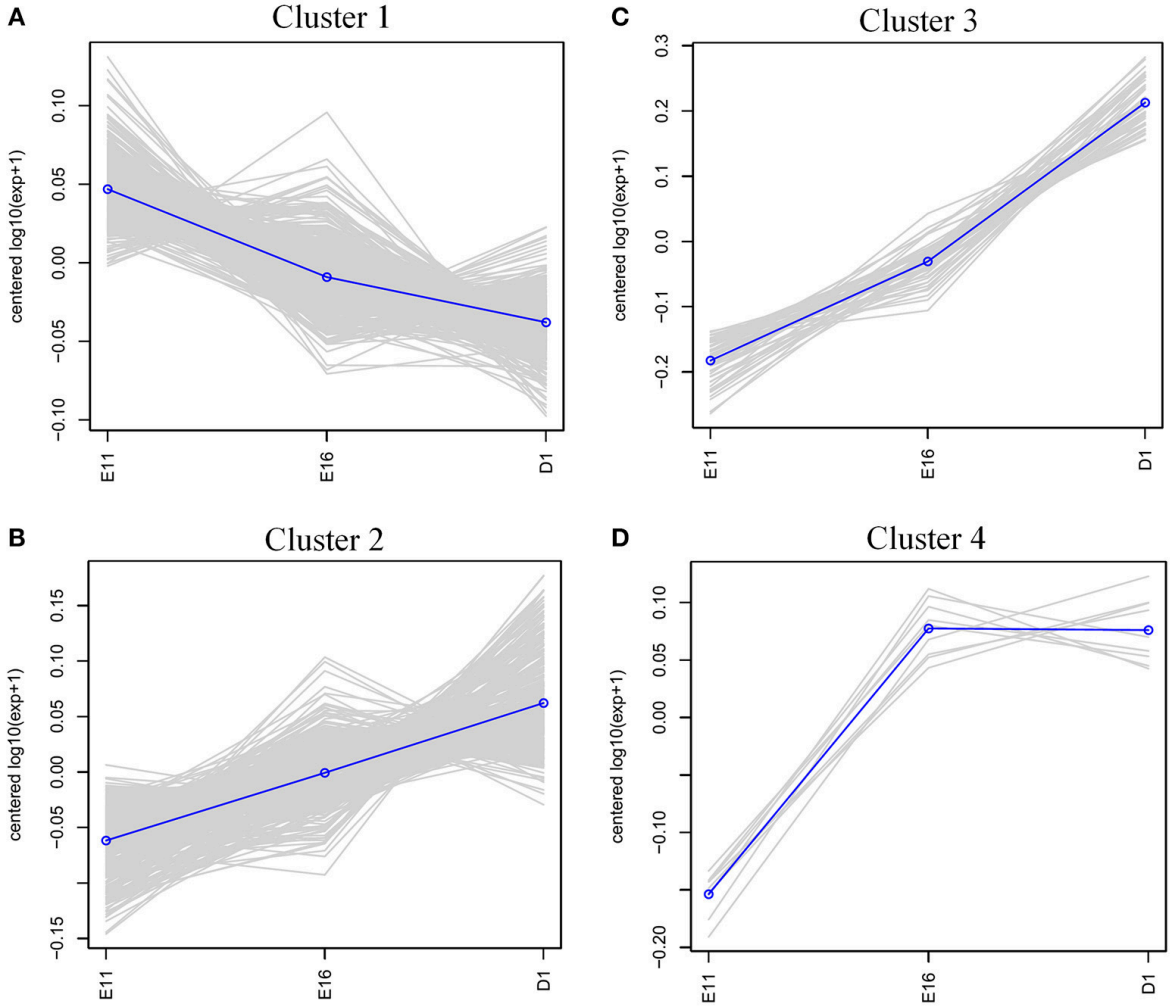
**Clustering analysis of the expression patterns of differentially expressed proteins. (A)** Cluster 1 (476 proteins); down-regulated consistently from E11 to D1. **(B)** Cluster 2 (358 proteins); up-regulated steadily from E11 to D1. **(C)** Cluster 3 (52 proteins); up-regulated sharply from E11 to D1. **(D)** Cluster 4 (9 proteins); up-regulated sharply from E11 to E16, but then no significant change at D1.

To characterize the expression patterns of differentially expressed proteins (fold change ≥ 1.2 or ≤ 0.8 and *p* < 0.05), cluster analysis was performed using hcluster. The heatmap of these differentially expressed proteins indicated that their expression patterns are similar in E16 and D1 (Figure S2). According to the expression pattern, these differentially expressed genes could be mainly grouped into four distinct clusters (Figure 3). The largest cluster (cluster 1, including 476 proteins) had a consistently overall pattern of down-regulation from E11 to D1 (Figure 3A). However, t cluster 2 (358 proteins) were up-regulated steadily from E11 to D1 and the 52 proteins of cluster 3 were up-regulated sharply from E11 to D1. The nine proteins of cluster 4 were up-regulated sharply from E11 to E16 but showed no significant change at D1 (Figure 3D).

### Western blot validation of iTRAQ data

**Figure 4:**
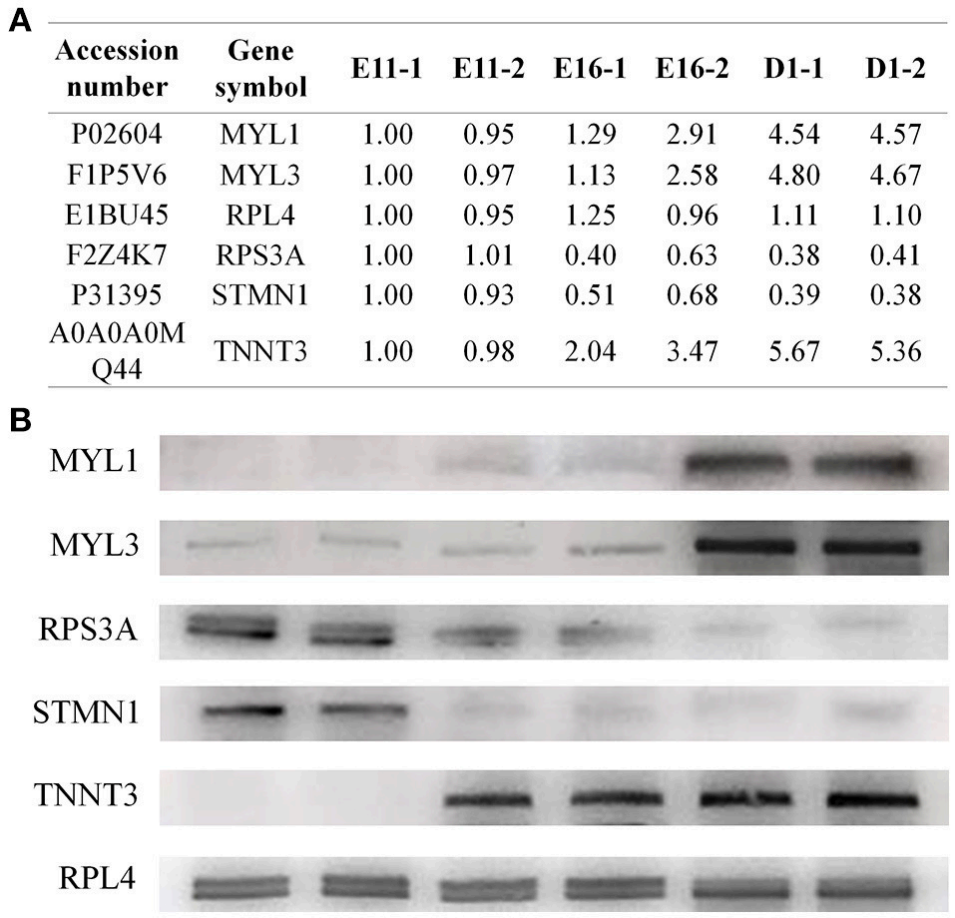
**Western blotting validation of the differentially expressed proteins. (A)** The proteome data for candidate proteins. **(B)** Western blotting results showed that the expression levels of MYL1, MYL3, and TNNT3 all increased, while RPS3A and STMN1 decreased from E11 to D1. The protein RPL4 was used as reference gene.

To confirm the iTRAQ results, we performed western blotting analysis for several candidate proteins, including MYL1 (myosin light chain 1), MYL3 (myosin light chain 3), RPS3A (ribosomal protein S3A), STMN1 (stathmin 1) and TNNT3 (troponin T3) (Figure 4A). The no obvious changes protein of RPL4 (ribosomal protein L4) was used as reference gene. The expression levels of MYL1, MYL3, and TNNT3 all increased from E11 to D1, while the RPS3A and STMN1 decreased during this same period (Figure 4B). The expression patterns of these proteins were consistent with our iTRAQ data.

### Interaction network of differentially expressed proteins

**Figure 5:**
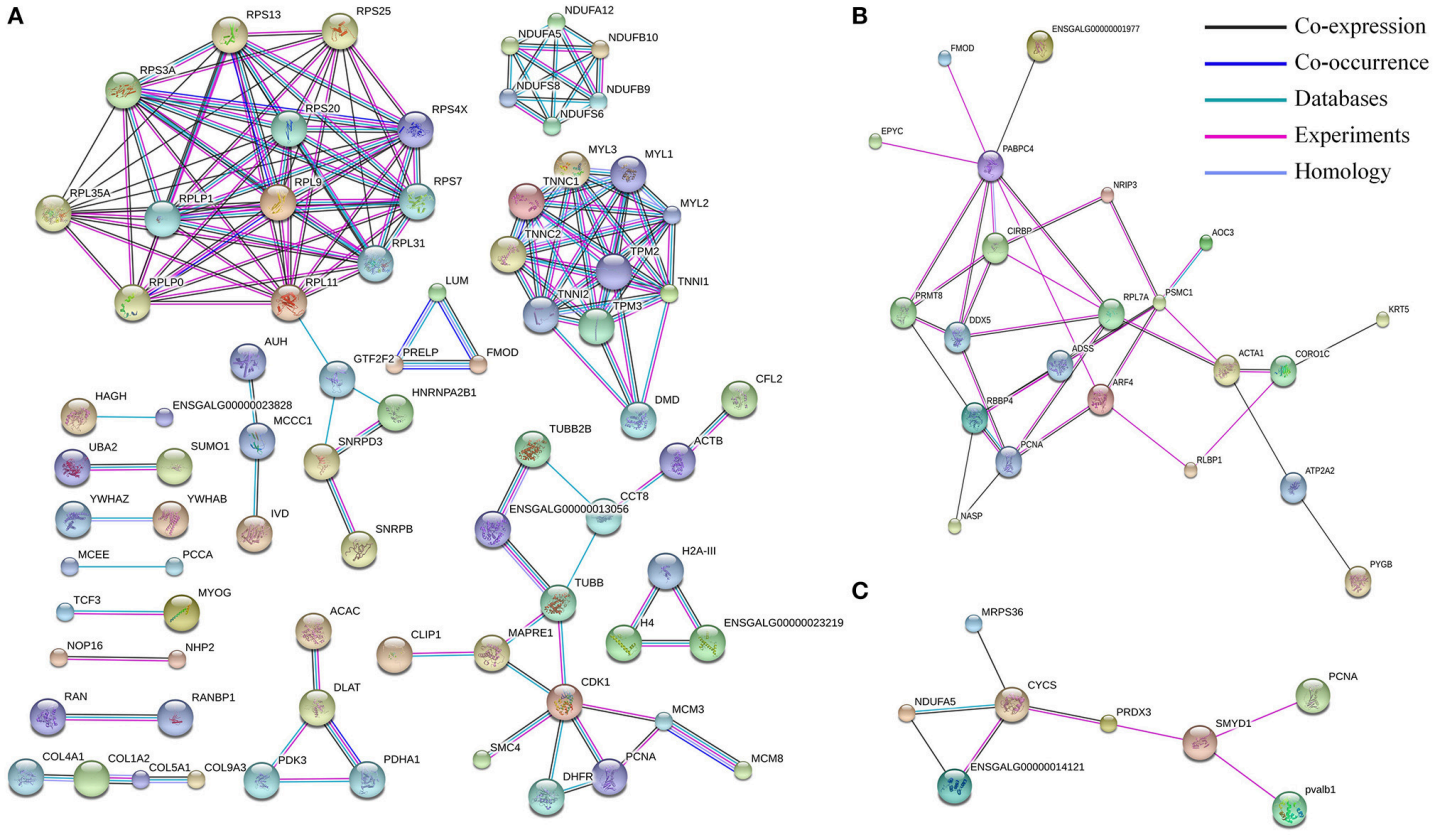
**The protein–protein interaction network of differentially expressed proteins in three comparisons: E11 vs. D1 (A)**, E11 vs. E16 **(B)** and E16 vs. D1 **(C)**. In this network, nodes represent proteins, and lines with different color represent the predicted different associations.

Proteins are usually not work alone, but rather interacted with each other to perform various functions. To explore the protein interaction networks altered in development of chicken embryonic skeletal muscle, differentially expressed proteins identified in this study were analyzed using STRING software. In E11 vs. D1 group, 396 of 465 differentially expressed proteins were detected in STRING, and 77 proteins were connected to nodes in the network (Figure 5A). Interestingly, we found interaction networks for ribosomal protein (RPS20, RPS25, RPLP1, and RPL9 etc.), muscle contraction (TNNC1, TNNC2, TNNI1, TNNI2, TPM2, TPM3, MYL1, MYL2, MYL3, and DMD), pyruvate metabolism (PDHA1, PDK3, and ACAC) and oxidative phosphorylation (NADH: ubiquinone oxidoreductase family, including NDUFA5, NDUFS6, and NDUFB9 etc.). In E11 vs. E16 group, 43 differentially expressed proteins were analyzed and 22 of them constituted an interaction network. This network was related to pathway of GTP and myosin binding and included ADSS, ARF4, PSMC1, ACTA1, and ATP2A2 (Figure 5B). In E16 vs. D1 group, 16 of 18 differentially expressed proteins were detected and eight of them constituted a network including NDUFA5, CYCS, PRDX3, SMYD1, and PCNA (Figure 5C).

### Interaction network of proteome and transcriptomes integrative analysis

**Figure 6:**
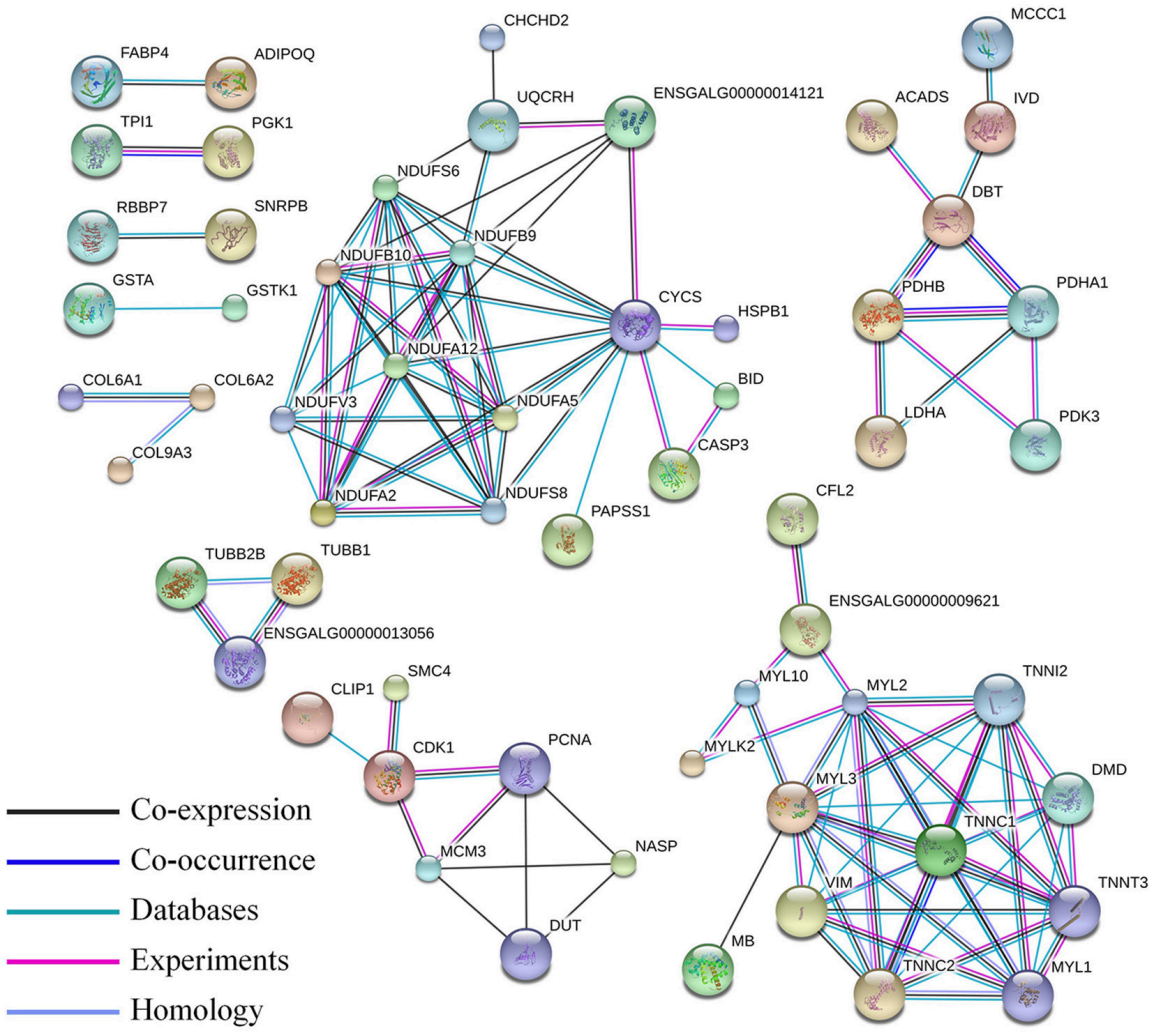
**Protein–protein interaction network of the differentially expressed proteins correlated with their mRNA expression level**. In this network, nodes represent proteins, and lines with different colors represent the predicted associations.

Skeletal muscle development in the chicken is a complex physiological process, and gene expression is regulated at multiple levels. Thus, we performed a proteome and transcriptomes integrative analysis to give us clues as to the levels of regulation for the differentially expressed proteins. When we compared our differentially expressed proteins with the differentially expressed mRNAs, 189 differentially expressed proteins were regulated in the same manner as their mRNAs (Table S6). The interaction between these proteins was also involved in the pathway of muscle contraction and oxidative phosphorylation (Figure 6).

**Figure 7:**
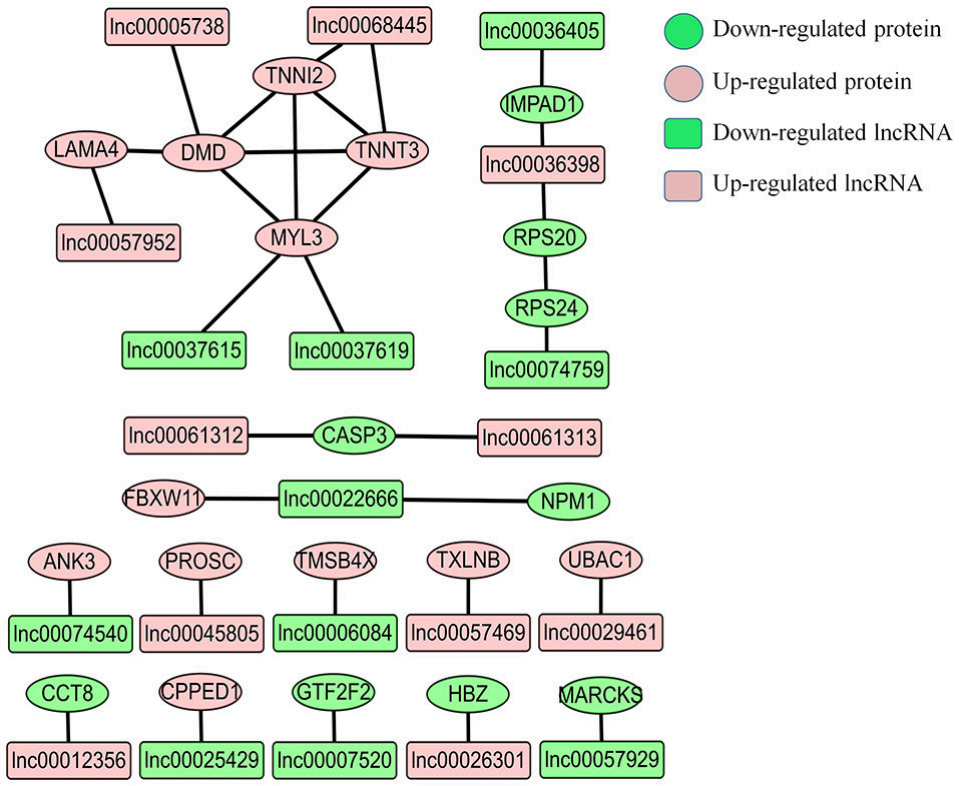
**Proteins-lncRNA network for comparison group of E11 vs. D1**. The predicted potential target genes of differentially expressed lncRNAs compared with corresponding differentially expressed proteins. The pink circles represent up-regulated proteins, and the green circles represent down-regulated proteins. The pink rectangles represent up-regulated lncRNAs, and the green rectangles represent down-regulated lncRNAs.

LncRNAs can regulate gene expression through cis-acting or trans-acting mechanisms (Bu et al., 2012; Han et al., 2012). We next integrated our lncRNA data with the proteomic data to construct an interaction network. We found that only a small fraction of differentially expressed proteins were from the target gene of corresponding differentially expressed lncRNAs. In E11 vs. E16 group and E16 vs. D1 group, only the MARCKS gene was identified as the target gene of lnc00057929, and KLHL15 gene was found as target gene of lnc00037615 and lnc00066198. For the E11 vs. D1 group, there were 21 differentially expressed proteins we found as target genes of 22 lncRNAs (Figure 7 and Table S7). Interestingly, four proteins (DMD, MYL3, TNNI2, and TNNT3) involved in muscle contraction were identified in this network, indicating that they may regulated by their corresponding lncRNAs (Figure 7).

## Discussion

Proteomic analysis is a powerful technique for investigating protein expression patterns, and has been used for chicken in several studies (Cao et al., 2012; Zhang et al., 2015; Desai et al., 2016; Gao et al., 2016; Li et al., 2016a,b). Skeletal muscle is an essential component of the animal body and its mass directly correlates with meat production (Güeller and Russell, 2010). Numerous studies have been identified proteome changes of skeletal muscle at different growth stages in chickens (Doherty et al., 2004; Teltathum and Mekchay, 2009; Liu et al., 2016b), pigs (Long et al., 2016; Zhang et al., 2016), cattle (Zhang et al., 2010), and rats (Spácilová et al., 2016). Embryonic myogenesis involves in a series of complex biological process, which is a major determinant of muscle mass (Buckingham, 2001). Chicken skeletal muscle also offers an excellent system for developmental proteomics. However, for chicken skeletal muscle, previous proteomic analyses concentrated on days post hatching. For example, Doherty et al. characterized the proteome of layer chicken breast muscle at specified time-points from 1 to 27 days after hatching (Doherty et al., 2004). Protein expression profiles were also performed in the breast muscle of Thai indigenous chickens at 0, 3, 6, and 18 weeks of age and Beijing-You chickens at ages 1, 56, 98, and 140 days (Teltathum and Mekchay, 2009; Liu et al., 2016b). In this study, we performed an iTRAQ-based proteomic analysis of chicken skeletal muscle at E11, E16, and D1, so we could focus on myogenesis during embryonic development. In the chick, fetal myoblasts are most abundant from E8 to E12, and undergo massive differentiation at E16 to E18 (Hartley et al., 1992; Stockdale, 1992).

During chick embryo development, muscles of the limbs are originated from the somite; progenitor cells migrated to their final destination in the limb at E11, and then quickly start to differentiate by the activation of the muscle determination factors MYOD, MYOG, and MRF4 (Bismuth and Relaix, 2010). We identified 3,240 proteins and 491 of them were differentially expressed between the three developmental ages in leg muscle. The greatest numbers of differentially expressed proteins was in the E11 vs. D1 (*n* = 465) group, and interaction work of these proteins were found clusters for ribosomal structural proteins (protein synthesis), muscle contraction (specific muscle function), pyruvate metabolism and oxidative phosphorylation (energy generation). These results could provide new clues for genetic regulation of embryonic myogenesis.

Ribosomal proteins usually constitute the ribosomal subunits, and involved in the cellular process of translation and protein biosynthesis. Ribosomal proteins have been reported that participated in regulation of mRNA translation during myogenesis (de Klerk et al., 2015). In breast muscle of Beijing-You chickens, ribosome-related functional modules were also found in interaction networks of the differentially expressed proteins at days 1, 56, 98, and 140 days (Liu et al., 2016b). We found that all these differentially expressed ribosomal proteins were down-regulated at D1. The mitochondrial oxidative phosphorylation system is responsible for providing the bulk of cellular ATP. More than 90% of the cells of ATP are synthesized by oxidative phosphorylation in mitochondria (Minai et al., 2008; Pejznochova et al., 2010). In this E11 vs. D1 group, the differentially expressed proteins NDUFA5, NDUFA12, NDUFB9, NDUFB10, NDUFS6, and NDUFS8 are all involved in oxidative phosphorylation and most were up-regulated at D1 (Figure 5A). This family of proteins was also represented in our interaction network of the 189 differentially expressed proteins that directly correlated with their corresponding mRNA levels (Figure 6).

In the muscle contraction pathway, 10 differentially expressed proteins from the E11 vs. D1 group were found involving in this pathway, including troponin subunits TNNC1, TNNC2, TNNI1, TNNI2, and tropomyosin subunits TPM2 and TPM3, as well as MYL1, MYL2, MYL3, and DMD (Figure 5A). Myosin light chains (MYL1, MYL2, and MYL3) are components of the myosin complex, and could act as a molecular motor to provide the energy for muscle contraction (Dominguez et al., 1998). The TNNC1, TNNC2, TNNI1, and TNNI2 are important components of troponin-tropomyosin complex, which has been found play a crucial role in the regulation of muscle contraction (Farah and Reinach, 1995). The DMD gene encodes dystrophin, that is essential for the development and organization of myofibers in skeletal and cardiac muscles (Muntoni et al., 2003; Ghahramani Seno et al., 2010). All these proteins were up-regulated at D1. Interestingly, the proteins interaction network in Figure 6 were also involved in the pathway of muscle contraction, including TNNC1, TNNC2, TNNI2, TNNT3, MYL1, MYL2, MYL3, and DMD etc. The proteins DMD, MYL3, TNNI2, and TNNT3 were also identified in our lncRNA-proteins interaction network (Figure 7). The TNNI2 and TNNT3 is the putative cis-target of lnc00068445; DMD is the putative cis-target of lnc00005738; MYL3 is the putative cis-target of lnc00037615 and lnc00037619. These four proteins may regulated by their corresponding lncRNA and together take part in the regulation of muscle development.

In conclusion, the present study characterized the proteins expression profile of chicken during embryonic skeletal muscle development by iTRAQ. A total of 491 differentially expressed proteins were identified at three different developmental groups. The differentially expressed proteins were mainly involved in the pathway of ribosome, muscle contraction, and oxidative phosphorylation. Several proteins that involved in the pathway of muscle contraction were found may regulated by lncRNA. Overall, the results can provide a more comprehensive insight into the regulation networks and biochemical pathways during embryonic skeletal muscle development in chicken.

## Author contributions

HO: performed the experiments, analyzed the data and wrote the manuscript. ZW and XC: Collected the samples and analyzed the data. JY and ZL: analyzed the data. QN: designed the study and reviewed the manuscript. All authors have read and approved the final manuscript.

## Funding

This research was supported by the Program for New Century Excellent Talents in University (NCET-13-0803) and the Foundation for High-level Talents in Higher Education of Guangdong, China.

### Conflict of interest statement

The authors declare that the research was conducted in the absence of any commercial or financial relationships that could be construed as a potential conflict of interest.

## Supplementary material

Figure S1 **KEGG analysis of differentially expressed proteins in E11 vs. E16 (A)**, E11 vs. D1 **(B)** and E16 vs. D1 **(C)**.

Figure S2 **Heatmap of differentially expressed proteins in three different development stages of embryonic muscle**.

Table S1 **The identified 3,250 protein list in chicken embryonic muscle by iTRAQ**.

Table S2 **GO analysis of 3,250 proteins in chicken embryonic muscle**.

Table S3 **Differentially expressed proteins in E11 vs. E16**.

Table S4 **Differentially expressed proteins in E11 vs. D1**.

Table S5 **Differentially expressed proteins in E16 vs. D1**.

Table S6 **Differentially expressed proteins correlated with RNA-seq**.

Table S7 **The lncRNAs target with differentially expressed proteins in E11 vs. D1**.

## Abbreviations

D1: 1 days post hatch
DEP: differentially expressed proteins
E11: embryonic age 11
E16: embryonic age 16
GO: gene ontology
iTRAQ: isobaric tags for relative and absolute quantification
KEGG: Kyoto Encyclopedia of Genes and Genomes
LncRNA: long non-coding RNA
XH: Xinghua chicken.
