# Supplementary figures and images for: Proteomic Analysis of Chicken Skeletal Muscle during Embryonic Development
Source: Front Physiol. 2017 May 8;8:281. doi: 10.3389/fphys.2017.00281 (PMC5420592; doi:10.3389/fphys.2017.00281)

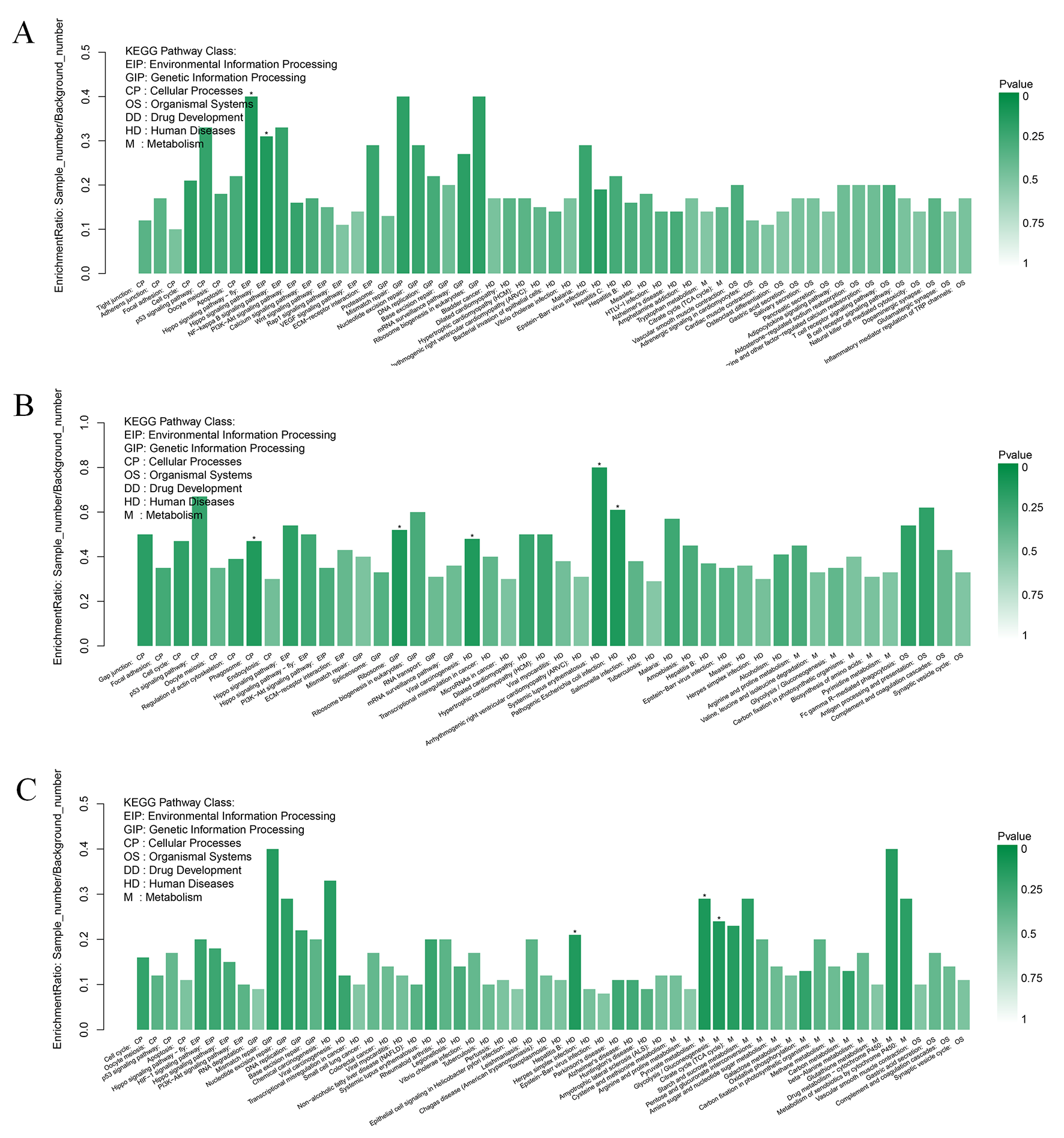

Supplement: Figure S1 — KEGG analysis of differentially expressed proteins in E11 vs. E16 (A), E11 vs. D1 (B) and E16 vs. D1 (C). [file Image1.TIF]

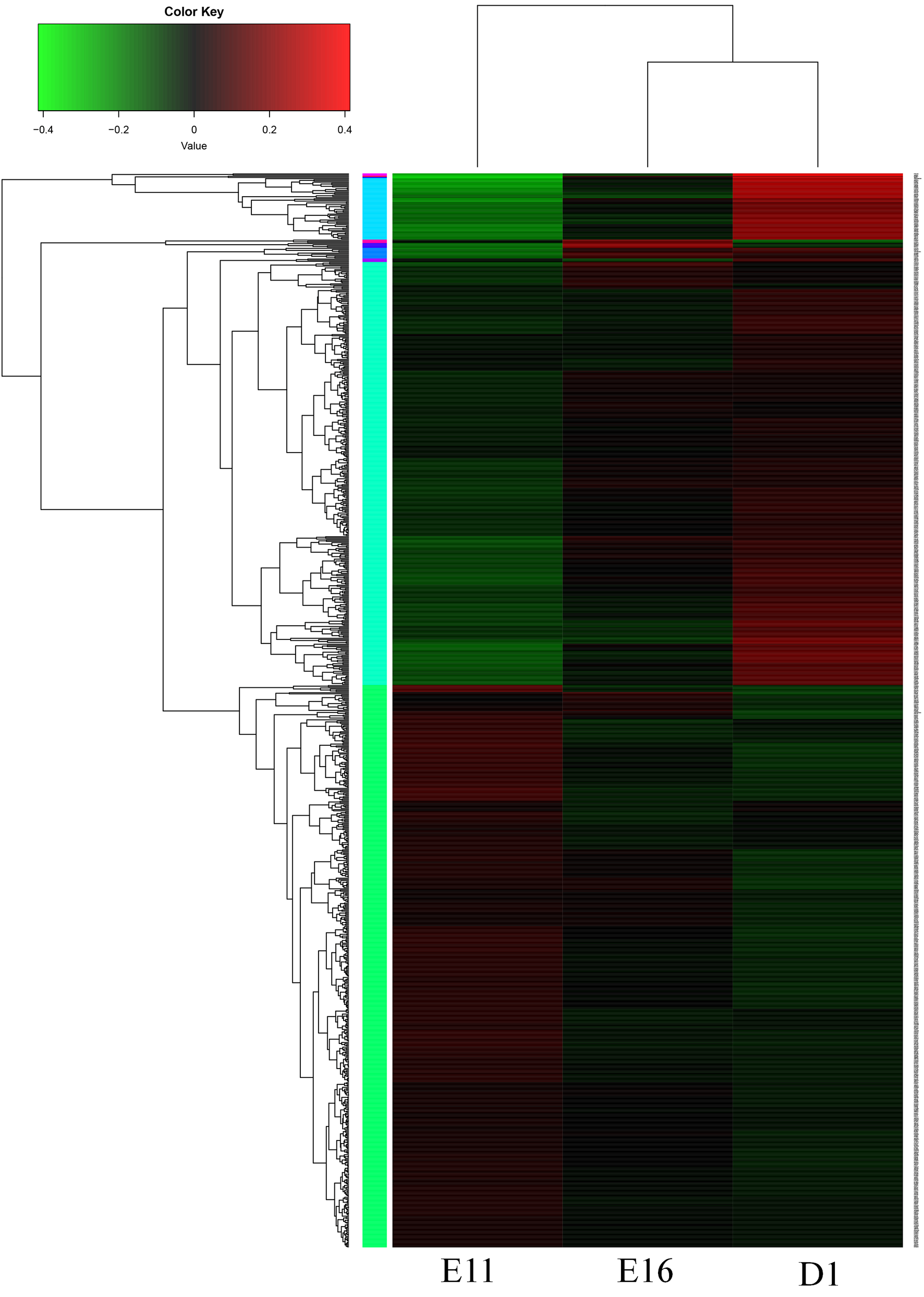

Supplement: Figure S2 — Heatmap of differentially expressed proteins in three different development stages of embryonic muscle. [file Image2.TIF]
